# Spin texture and chiral coupling of circularly polarized dipole field

**DOI:** 10.1515/nanoph-2022-0581

**Published:** 2023-01-03

**Authors:** Yu Shi, Hong Koo Kim

**Affiliations:** Department of Electrical and Computer Engineering and Petersen Institute of NanoScience and Engineering, University of Pittsburgh, Pittsburgh, PA 15261, USA

**Keywords:** chiral coupling, dipole near-field wave, plasmonic waveguides

## Abstract

We show that a circularly polarized electric dipole harbors a near-field concentrated wave which orbits around with an energy flux significantly larger (five orders of magnitudes at ∼1 nm radial distance) than far-field radiation. This near-field wave is found to carry transverse spins and reveal skyrmion spin texture (Néel-type). By performing electromagnetic analysis and numerical simulation, we demonstrate chiral extraction of a near-field rotational energy flux: the confined energy flow is out-coupled to surface plasmons on metal surface, whose curvature is designed to provide orbital angular momentum matched to spin angular momentum of dipole field, that is, to facilitate spin–orbit interaction. Strong coupling occurs with high chiral selectivity (∼113) and Purcell enhancement (∼17) when both linear and angular momenta are matched between dipole field and surface plasmons. Existence of a high-intensity energy flux in the deep-bottom near-field region (*r* ∼ 1 nm) opens up an interesting avenue in altering fundamental properties of dipole emission. For example, extracting ∼1% of this flux would result in enhancing spontaneous emission rate by ∼1000 times.

## 1 Introduction

Emission from an oscillating dipole is an elemental form of electromagnetic (EM) radiation and has been extensively studied since the dawn of quantum theory. Unlike the well-established far-field regime, it is relatively recent that the near-field regime started revealing a variety of novel phenomena and has drawn increasing attention [1–7]. Near-field experiments commonly involve a dense object as a probe, which is brought into close proximity of a dipole to enable extraction of an otherwise-nonradiating energy flux [8]. Here a dense object normally refers to metal or dielectric particles of nano- to micro-scale and serves as an antenna that reradiates incident light into characteristic directions. As such, the resolution of this technique is governed by probe dimensions, and its practical limits are typically of 10 nm order or larger. Moreover, the measurement output is basically a convolution of a probe response function and an original input signal. In other words, the overall result depends on details of sub-processes, such as how input field couples to probe field and how they radiate away into far-field.

Here we note the following outstanding issues: the nature of emitter-to-probe coupling widely varies depending on details (material, structure, geometry, configuration, etc.); and the underlying mechanisms are not clearly established yet, although major advancements have recently been made in understanding the subject phenomena [9–13]. Given the current state of our knowledge base of this field, it is highly desired to elucidate governing principles at a fundamental level such that they can explain a variety of different-looking experimental observations in a unified framework and even can predict ultimate performances.

In this work we address these issues by investigating the deep-near-field regime (e.g., <<10‐nm distance from a dipole emitter operating at optical frequency) and by exploring the governing mechanisms of out-coupling of dipole field to surface plasmons. Specifically, we directly calculate dipole fields in all regimes referring to explicit analytical expressions, which list multiple terms of different orders in distance dependence and therefore readily distinguish near-field evanescent waves from a far-field radiating one. In an alternative approach, dipole fields could also be analyzed by employing angular spectrum analysis, in which fields are transformed into wave-vector space and then classified into evanescent or far-field-propagating waves [8]. Compared to this transform method, our approach offers an advantage that we can directly map out all important field variables on a physical (space-time) domain.

We focus on analyzing energy flow (Poynting vector) and spin angular momentum (SAM) distributions, both calculated from electric and magnetic fields discussed above. Being governed by a conservation principle, SAM vector ***S*** plays an important role in analyzing dynamical evolution of EM phenomena. Poynting vector ***P*** relates to momentum density vector ***P***/*c* ^2^, where *c* is the speed of light, and to wavevector ***k***; and both vectors abide by conservation principles. In evanescent waves carrying transverse spins, the relationship between transverse SAM and Poynting vectors is known to resemble the duality of electric and magnetic fields [13]: for example, the curl operation of ***P*** results in ***S***, vice versa, similar to the curl relations of ***E***- and ***H***-fields in Maxwell’s equations. This suggests the boundary conditions for ***S*** and ***P*** would be similar to those for ***E***- and ***H***-fields, except that the tangential and normal boundary conditions for ***P*** and ***S*** will swap their expressions because of their cross-product relationship in terms of field vectors (***E*** and/or ***H***). Overall, the tangential components of spin angular momentum (***S***) and linear momentum (***k***) are expected to satisfy conservation requirements, which usually manifest in different forms such as continuity or phase-matching condition at an interface.

Based on the classical electromagnetic analysis outlined above, we discovered the followings: existence of a near-field energy flow circling around in the azimuthal direction; this rotational energy flux rapidly grows proportional to the inverse cube of distance *r*; at *r* = 1 nm, for example, it becomes five orders of magnitudes greater than far-field radiation; the near-field wave carries transverse spins with skyrmion-like spin texture [14–18]; the spin vector orients upward at the north pole, flips down at equatorial region and then flips up again around the south pole. We also designed a plasmonic coupler structure [19–28] that demonstrates chiral extraction of near-field rotational energy; a U-shape Ag nanowire waveguide is simulated for transfer coupling of dipole field to surface plasmons; the radius of curvature of a U-shape part is designed to provide orbital angular momentum (OAM) matched to the SAM of an incident photon; maximum chiral coupling occurs when the conservation conditions are met for both angular and linear momenta.

## 2 Spin texture and energy flow distribution

**Figure 1:**
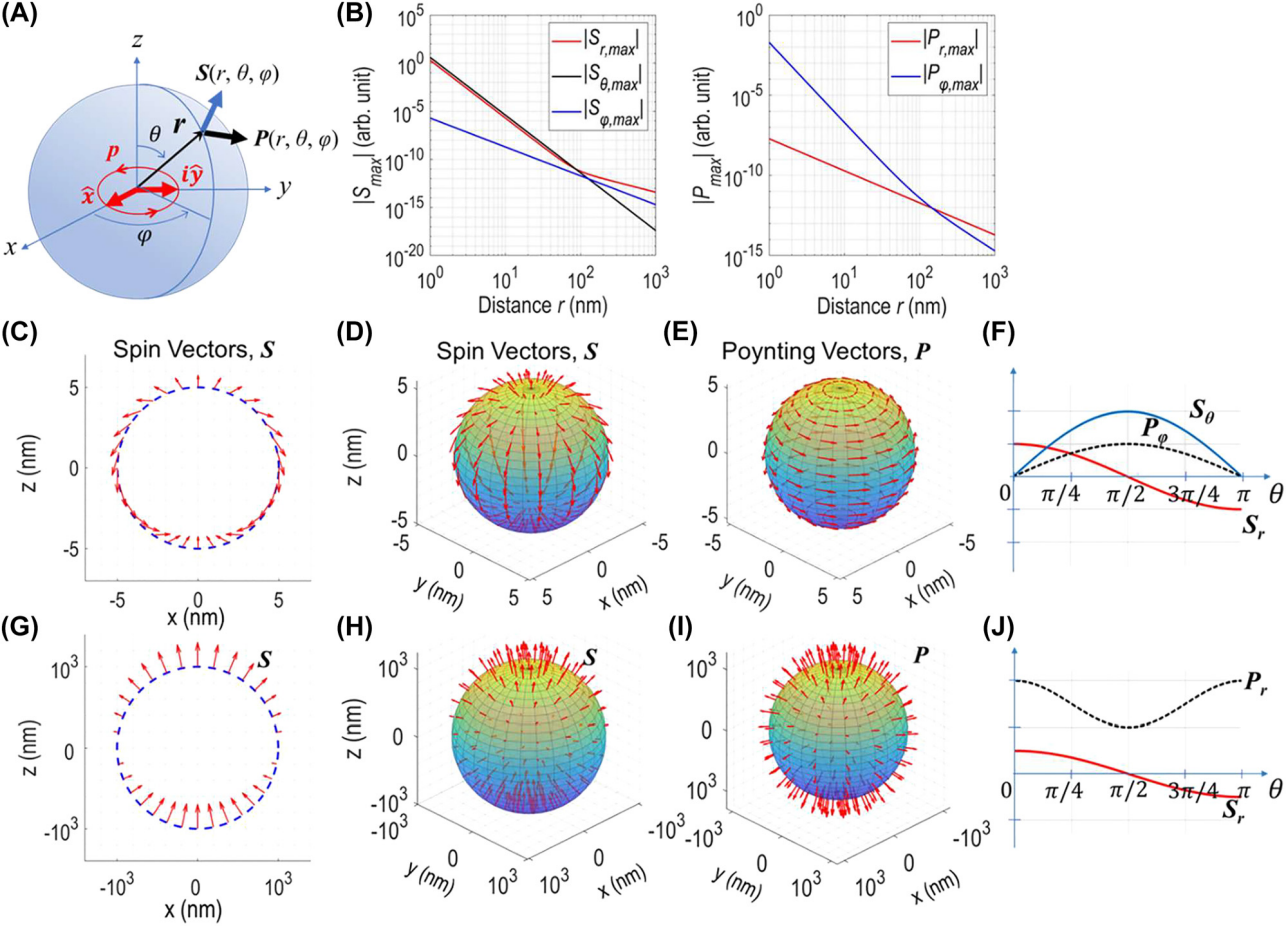
Spin texture and energy flow distribution of an electric dipole with circular polarization. (A) A dipole moment $p=\frac{p_{0}}{\sqrt{2}}\left(\hat{x}+i\hat{y}\right)$ placed at the origin of spherical coordinates; the dipole axis is along the *z*-direction. (B) The distance (*r*) dependence of spin density and energy flow: the magnitudes of spin vector (*S*_*r*_, *S*_*θ*_ and *S*_*φ*_: left panel) and Poynting vector (*P*_*r*_ and *P*_φ_: right panel). The angular (*θ*, *φ*) distribution of spin vector (C and D) and Poynting vector (E) calculated at *r* = 5 nm. Spin vector plot on the circle of a cross-section of a sphere (C). Spin vectors orient orthogonal to energy flow direction revealing transverse spins at near field. (F) Spin (*S*_*r*_, *S*_*θ*_) and Poynting (*P*_*φ*_) vector distributions plotted as a function of polar angle (*θ*). Note a skyrmion texture (C and D) of spin vector distribution: spin flips once when scanned along the polar angle direction (*θ*: from 0 to π). The angular (*θ*, *φ*) distribution of spin vector (G and H) and Poynting vector (I) calculated at *r* = 1 μm. Spin vector plot on the circle of a cross-section of a sphere (G). Spins orient parallel (antiparallel) to energy flow direction in the polar regions, revealing longitudinal spins at far field. (J) Spin (*P*_r_) and Poynting (*S*_r_) vector distributions plotted as a function of polar angle.

Consider an electric point-dipole with dipole moment $p=\frac{p_{0}}{\sqrt{2}}\left(\hat{x}+i\hat{y}\right)$ placed at the origin of spherical coordinates: a polarization vector lies on the *x*–*y* plane and rotates about the dipole axis (the *z*-axis) (Figure 1A). After some work, we obtain electric and magnetic fields (***E*** and ***H***) expressed in SI units [29] (Supporting Information Section S1).

(1) $$\begin{array}{ll} E\left(r\right) & =\frac{p_{0}}{4\sqrt{2}\pi \varepsilon }e^{\mathrm{ikr}}{\text{e}}^{\text{i}\varphi }\left[2\left(\frac{1}{r^{3}}-\frac{ik}{r^{2}}\right)\sin\theta \;\hat{r}\right.\\ & \;\left.+\left(\frac{k^{2}}{r}+\frac{-1}{r^{3}}+\frac{ik}{r^{2}}\right)\left(\cos\theta \;\hat{\theta }+i\;\hat{\varphi }\right)\right] \end{array}$$

(2) $$H\left(r\right)=\frac{p_{0}}{4\sqrt{2}\pi \sqrt{\mu \varepsilon }}e^{\mathrm{ikr}}{\text{e}}^{\text{i}\varphi }\left(\frac{k^{2}}{r}+\frac{ik}{r^{2}}\right)\left(-i\hat{\theta }+\cos\theta \hat{\varphi }\right)$$

where *r* is a distance from the dipole at the origin; *k* is a propagation constant; and $\hat{r}$ , $\hat{\theta }$ and $\hat{\varphi }$ denote unit vectors of spherical coordinates (*r*, *θ*, *φ*). *ɛ* is the permittivity; and *μ* is the permeability of ambient medium.

Spin angular momentum density ***S*** is defined as $S\left(r\right)=\frac{1}{4\omega }\left[\varepsilon \cdot Im\left(E^{*}\times E\right)+\mu \cdot Im\left(H^{*}\times H\right)\right]$ [13], where *ω* is the angular frequency of time harmonic oscillation *e* ^−*iωt*^; Im denotes the imaginary part of a complex number. SAM density is determined as:

(3) $$\begin{array}{ll} S\left(r\right) & =\frac{p_{0}^{2}}{64\pi^{2}\omega \varepsilon }\left[\left(\frac{1}{r^{6}}+\frac{2k^{4}}{r^{2}}\right)\cos\theta \hat{r}+\frac{2}{r^{6}}\sin\theta \hat{\theta }\right.\\ & \;\left.+\frac{k^{3}}{r^{3}}\sin2\theta \hat{\varphi }\right] \end{array}$$

The magnetic-field’s contribution to this spin density ***S*** widely varies depending on radial distance: null in the near-field; relatively minor in the intermediate field; and the same amount as the *E*-field’s contribution in the far-field regime (Supporting Information Section S1).

An energy flow density, i.e., time-averaged Poynting vector ***P***, is defined as ½ Re(***E*** * × ***H***) and is determined as

(4) $$\begin{array}{ll} P\left(r\right) & =\frac{p_{0}^{2}}{64\pi^{2}\varepsilon \sqrt{\mu \varepsilon }}\left[\frac{k^{4}}{2r^{2}}\left(3+\cos2\theta \right)\hat{r}\right.\\ & \;\left.+2\left(\frac{k^{3}}{r^{3}}+\frac{k}{r^{5}}\right)\sin\theta \hat{\varphi }\right] \end{array}$$

The radial-distance dependence of the *r-*, *θ-* and *φ-*components of ***S*** and ***P*** vectors is plotted for *r* in the range of 1 nm to 1 μm at 633 nm wavelength (Figure 1B). The spin vector in near-fields contains the azimuthal component (*S*_φ_): see Eq. (3). But its size is much smaller (by three orders of magnitude at *r* = 5 nm) than the radial (*S*_r_) and polar-angle (*S*_θ_) components: see Figure 1B (left). As such, it is not clearly visible in the 3D plot of Figure 1D.

Each arrow in Figure 1C–E and G–I, indicates the magnitude and orientation of spin or Poynting vector on a local reference frame (*θ*, *φ*) on sphere surface. At near field, spin vectors align along the dipole axis in the polar regions (*θ* = ∼0° or ∼180°) and orient to the polar angle direction in the equatorial region (*θ* = ∼90°). To help visual understanding of spin vector orientations in both hemispheres, Figure 1C shows the near-field spin vector distribution plotted on the circle of the cross-section (cut-through the center) of the sphere. The spin vector orients to the +*z*-direction at both north and south poles, and it flips to the −*z*-direction at the equator with its size twice of that at poles. Figure 1G shows the far-field spin vector distribution plotted on the circle of the center cross-section of the sphere. The spin vector orients to the +*z*-direction in both polar regions and reduces to zero in the equatorial region.

Poynting vectors orient to the azimuthal direction: see Figure 1B (right) and 1E calculated at *r* = 5 nm. The orthogonal relation of ***S*** and ***P*** vectors confirms the dipole field carries transverse spins in the near-field regime.

Transverse spins are intrinsic to evanescent waves, and their three unit-vectors form a right-handed triad: $\hat{S}=\hat{P}\times \hat{n}$ , where $\hat{S},\hat{P}$ and $\hat{n}$ represent the orientation of spin and momentum (Poynting) vectors and evanescent direction, respectively [10, 11]. This relation dictates spin-momentum locking and manifests time-reversal symmetry.

At far field, both spin and Poynting vectors become orientating to the radial direction: energy flows mostly towards the dipole axis (the ±*z*-directions) while spins align parallel (antiparallel) to the energy flow direction in the northern (southern) hemisphere (Figure 1G–I, calculated at *r* = 1 µm). This parallel/antiparallel relation of ***S*** and ***P*** vectors indicates that a time-reversal symmetry breaking occurs in the far-field regime of a circularly polarized dipole.

The spin vector distribution in the near-field regime shown above reveals an optical skyrmion, specifically, of Néel-type [14–18]: spin flips once along the polar angle direction on a local reference frame when traversed from the North pole to the South pole on sphere surface. Note here that the spin texture is defined on sphere surface, and the spin-flip count induced by curvature itself is not taken into account in this count. This skyrmion spin texture is maintained independent of radial distance as long as they remain within the near-field regime (*kr* << 1).

## 3 Near-field stored energy flux versus far-field radiation power

The near-field distributions of spins and Poynting reveal another novel feature (Figure 1C–E): existence of a confined wave that circles around a dipole with its intensity steeply rising (*r* ^−5^ order) for closer to a dipole.

The total flux of this stored energy flow is determined as (Supporting Information Section S2): $\int P\left(r\right)\cdot dA=\frac{\omega }{48\pi^{2}\varepsilon }\frac{p_{0}^{2}}{r_{1}^{3}}$ . Here the area of the integral is: radius *r* from *r*_1_ to ∞; polar angle *θ* from 0 to π. Let us assume: *p*_0_ = 1 nm · *e* for a dipole moment, where *e* is electron charge [30]; and 633 nm wavelength in free space. The total energy flux is calculated to be 6.6 × 10^−7^ J/s (or 2.1 × 10^12^ photons/s) when assuming the lower limit of integral *r*_1_ = 3 nm.

Let’s compare this energy flux with the total radiation power (Supporting Information Section S3): $\int P\left(r\right)\cdot dA=\frac{\omega k^{3}}{12\pi \varepsilon }p_{0}^{2}$ . Assuming the same parameter values as above, this far-field radiation is calculated to be 2.2 × 10^−10^ J/s. This amount corresponds to spontaneous emission rate of 7.0 × 10^8^ s^−1^ or emission lifetime of 1.4 ns.

The ratio of the near-field trapped energy flux to the radiation flux is $\frac{1}{4\pi {\left(kr_{1}\right)}^{3}}$ . At *r*_1_ = 3 nm (*kr*_1_ = 0.03 for *λ* = 633 nm), for example, this trapped energy flux is ∼3000 times greater than that of the radiative component; at *r*_1_ = 1 nm, it becomes ∼8 × 10^4^ times.

This finding, existence of a high-intensity energy flux in the deep bottom near-field (*r* of ∼1 nm) region, opens up an interesting avenue in altering fundamental properties of dipole emission. A fraction of this large energy flux would be far greater than the radiation power. Extracting such a large amount of energy flux out of a dipole would result in major enhancement of spontaneous emission rate. For example, extracting ∼1% of this flux at *r* ∼ 1 nm would result in enhancing the spontaneous emission rate by ∼1000 times.

## 4 Near-field confined wave: conformal relation to surface plasmons

The time evolution and spatial distribution of a near-field-confined traveling wave are analyzed. Let’s consider the case of *θ* = 90° at distance *r*_o_ (*kr*_o_ << 1). The *E*-field component responsible for ***S*** (***r***) is: $E\left(r,t\right)\propto e^{-i\omega t}{\text{e}}^{\text{i}\varphi }\left(2\hat{r}-i\hat{\varphi }\right)$ . The *E*- and *H*-field terms responsible for ***P*** (***r***) are: $E\left(r,t\right)\propto e^{-i\omega t}{\text{e}}^{\text{i}\varphi }\hat{r}$ ; $H\left(r,t\right)\propto e^{-i\omega t}{\text{e}}^{\text{i}\varphi }\hat{\theta }$ .

**Figure 2:**
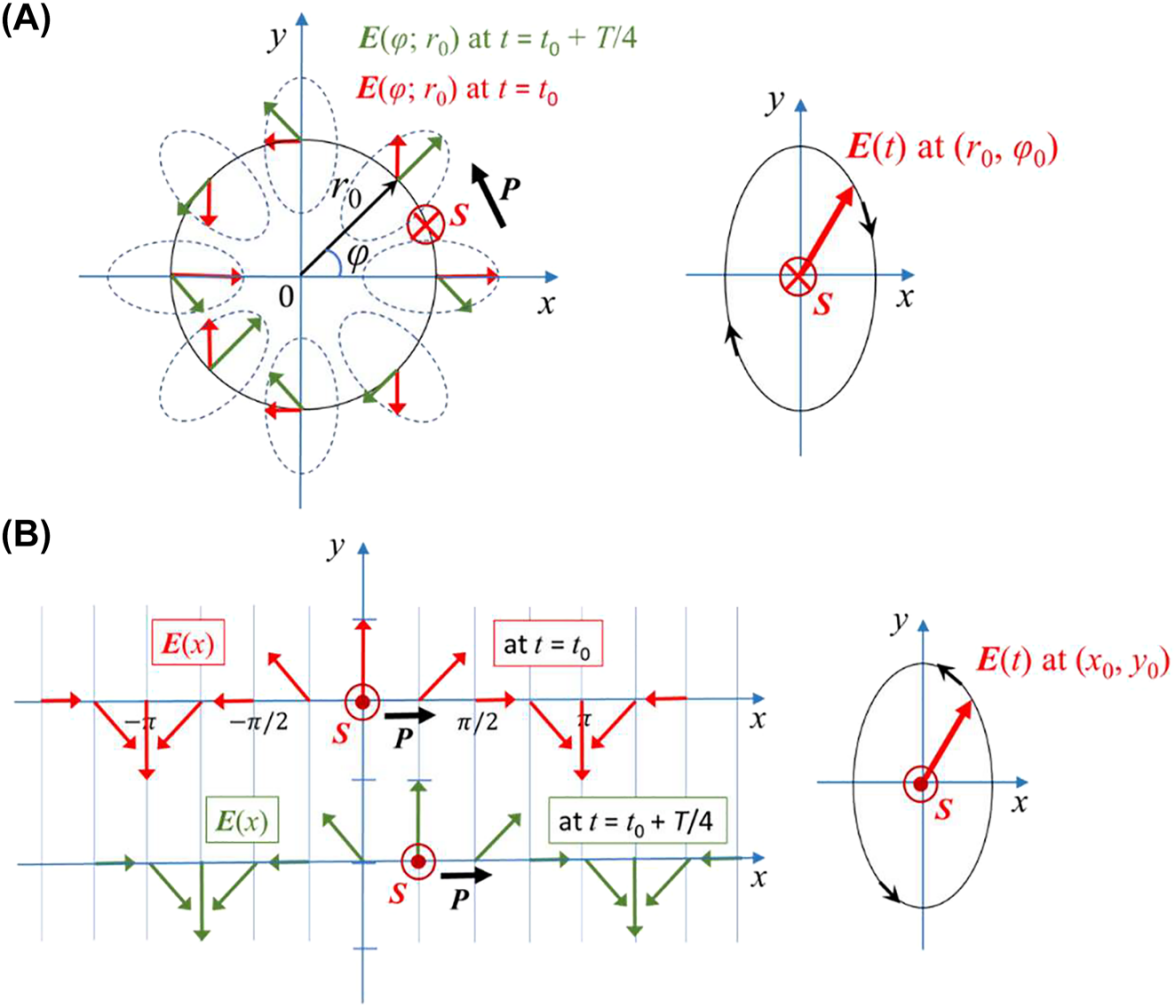
Spatial distribution and time evolution of electric fields with transverse spins. (A) Circularly polarized dipole field for *θ* = 90°. The spatial distribution of *E*-vector orientation at a given time point (left panel). The green arrows denote *E*-vector orientation and the red arrows are after a *T*/4-time lapse, where *T* is the period of oscillation. *E*-vector rotates counter-clockwise advancing its phase to the +*φ*-direction. *E*-vector at a given location elliptically rotates along the clockwise orientation (right panel). Spin vectors orient to the transverse direction (the −*z*-direction) and Poynting vectors to the azimuthal direction (the +*φ*-direction). (B) Surface plasmon field on a planar metal surface. At a given time point, *E*-vector rotates clockwise advancing its phase to the −*φ*-direction (left panel). *E*-vector at a given location rotates counter-clockwise (right panel). Spin vectors orient to the transverse direction (the +*z*-direction) and Poynting vectors to the propagation direction (the +*x*-direction).

The *E*-vector elliptically rotates on the *r*–*φ* plane with angular frequency *ω*, generating spins that orient to the polar angle direction $\hat{\theta }$ . (Recall that the *H*-field in the near-field regime does not contribute to ***S***.) The *E*-field phase also advances along the *φ*-direction while rotating around the dipole axis. This phase change in the azimuthal direction corresponds to geometric phase and explicitly appears in the *E*-field and *H*-field expressions: e^i*φ*^ in Eqs. (1) and (2). In this cycloidal process, *E*-vector makes a 2π rotation per oscillation, clockwise seen from the +*z*-side while looking down toward the −z-direction (Figure 2A right), while circling around the dipole, counter-clockwise seen from the +*z*-side while looking down toward the −*z*-direction (Figure 2A left).

Poynting vectors orient to the azimuthal direction ( $\hat{P}=\hat{\varphi }$ ): recall that ***P*** represents a time-averaged energy flow and is determined as a vector product of the *H*-field and the *E*-field component that is in-phase with the *H*-field. Spin vectors orient orthogonal to energy flow vectors $\left(\hat{S}=\hat{\theta }\right)$ forming transverse spins; both vectors obey the spin-momentum locking principle $\hat{S}=\hat{P}\times \hat{n}$ .

The electrodynamics of this near-field confined wave resembles surface plasmon (SP) propagation on a planar interface [31, 32]. Figure 2B shows SP fields propagating along the +*x*-direction: $E\left(r,t\right)\propto e^{-i\omega t}{\text{e}}^{\text{i}k_{x}x}\left(E_{y0}\hat{y}-iE_{x0}\hat{x}\right)$ ; $H\left(r,t\right)\propto e^{-i\omega t}{\text{e}}^{\text{i}k_{x}x}\hat{z}$ . The *E*-field rotates on the (*x*, *y*) plane and generates spins that orient to the *z*-direction. A conformal relation is found to exist between the two coordinate systems. For example, consider a transformation *z* ↦ e^*iz*^ = e^*ix*−*y*^ where *z* denotes a complex number $\left(\right.x+iy$ ). This maps a rectilinear coordinate (*x*, *y*) patch (−*π* < *x* < *π*; −∞ < *y* < ∞) into a polar coordinate (*r*, *φ*) patch (0 < *r* < ∞; 0 < *φ* < 2*π*). This conformal mapping preserves local geometry, that is, angles between coordinate lines in an infinitesimal space. Since spin vector represents the angular momentum of rotating *E*-field vector, spin angular momentum is conserved in this conformal mapping.

The strong confinement of dipole near-fields observed in this work is consistent with a recent report on understanding light–matter interactions with photonic quasiparticles [33]. Photonic quasiparticles are quantized time-harmonic solutions of Maxwell’s equations in an arbitrary inhomogeneous and dispersive medium, e.g., radiations from bound electron emitters. Certain types of photonic quasiparticles are known to confine EM fields down to the scale of a few nanometers, e.g., surface plasmons in 2D materials. The conformal relation observed in the present work suggests that the near-fields of a rotating dipole might be viewed as photonic quasiparticles and potentially offer an interesting venue to explore introducing quantum mechanical concepts to classical electromagnetic analysis.

## 5 Energy flow vortex and photonic orbitals

**Figure 3:**
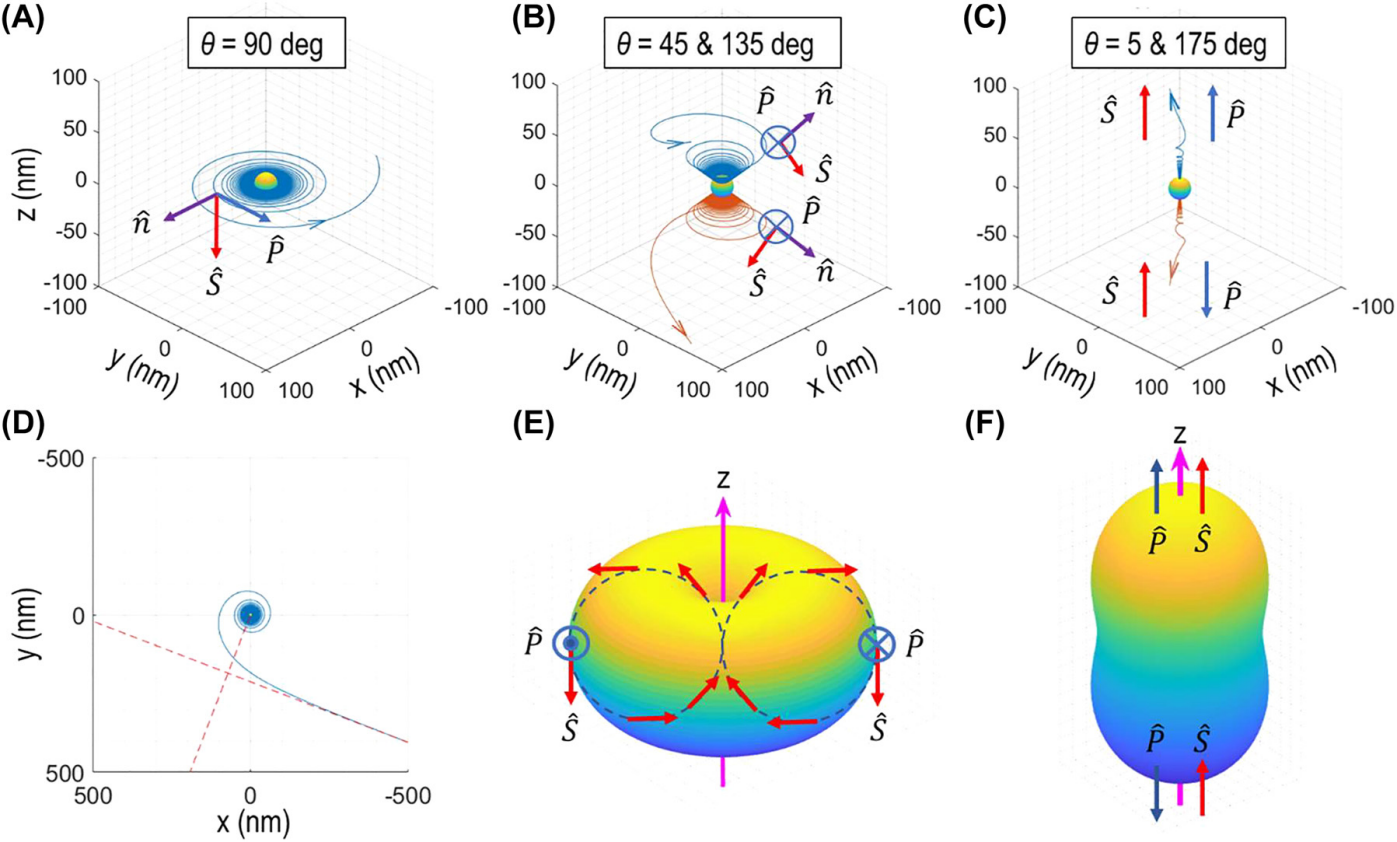
Energy flow lines and Poynting vector distributions. Trajectories calculated for three different polar angles: (A) *θ* = 90°; (B) *θ* = 45 or 135°; and (C) *θ* = 5 or 175°. (A–C) Plotted for *r* of 10–100 nm. The inner-most part of a trajectory (shown with a sphere: *r* < 10 nm) is excluded in this plot in order to reduce computational load: the flow lines make a large number of windings at near field (e.g., ∼10^7^ for *r* of 5–10 nm). The densely spaced vortex profiles are not clearly resolved in these plots. Spins orient orthogonal to energy flow direction (transverse spins) at near field. The three unit-vectors (spin, energy flow, and evanescent direction) form a right-handed triad $\hat{S}=\hat{P}\times \hat{n}$ . (D) An energy flow trajectory plotted on an equatorial plane (*θ* = 90°): a flow line spirals out to far field asymptotically approaching a straight-line exit. The orbital angular momentum value ranges from 0 at *kr* = 0 to *ℏ* at *kr* = 1.03 (marked with a red circle) and 2*ℏ* at *kr* = ∞. Note that the asymptotic line at far field is displaced from a dipole by distance *d* = *λ*/π. (E and F) The angular (*θ*, *φ*) dependence of energy flow distributions calculated at near field (*kr* << 1) (E) and at far field (F). The arrows denote spin angular momentum (red) and Poynting (blue) vectors at the locations where the likelihood of finding a photon is maximal.

The trajectory of an energy flow is governed by the orientation of Poynting vector at each point and can be plotted by integrating the unit vector over time-parameter space [7]. Energy flow lines are calculated for different polar angles (Figure 3A–C): *θ* = 5 and 175°; 45 and 135°; and 90°. The flow lines form a vortex of numerous turns in the near field; gradually spiral out in the mid-field; and asymptotically approach a straight-line exit at far field. The relation of ***S*** and ***P*** vector orientations evolves from being transverse in the near field regime to longitudinal at far field.

For the case of *θ* = 90°, the vortex profile is comprised of a large number of orbits in the near field region (Supporting Information Section S4). For example, the winding number is estimated to be 2 × 10^8^ for radial distance *r* of 3–5 nm; 1 × 10^7^ for *r* of 5–10 nm. This densely spaced spiraling trajectory is consistent with the observation that Poynting vectors at near field orient predominantly to the azimuthal direction (Figure 1B, right).

Quantization of light in free space is a long-established concept, and their quantum mechanical calculation is known to well converge to classical EM analysis, especially in far-fields. A rotating dipole is a good model of an atom emitting a photon during relaxation from an excited state to a ground state: this model can serve as a testbed to validate the convergence of both analyses. As shown above, the dipole emission rate calculated from a far-field analysis is found to be in good agreement with a quantum mechanical calculation of spontaneous emission rate of an atom [33].

The far-field radiation from a rotating electric dipole is known to carry angular momentum (AM), and their orbital and spin AM distributions have been reported [34, 35]. For example, Khrapko’s work [34], performed by classical electrodynamics analysis, revealed that the OAM flux in far-fields is twice of the SAM flux, being consistent with our observation (see below) that the OAM value in far-fields is bounded to two times (2*ℏ*) of SAM value (*ℏ*). In the near-field regime (*kr* << 1), the OAM becomes zero, and only the SAM remains. Gough’s EM analysis [35] also shows that the ratio of an energy flux to an OAM flux is equal to *ω* (angular frequency), corresponding to the well-known relationship between photon energy (*ℏω*) and angular momentum (*ℏ*).

In this work, we postulate that the spiraling energy-flow trajectories with an off-shifted exit line in far-fields can be associated with a notional photon that carries OAM. Here it should be noted that the energy flow lines should not be interpreted as a flight line of a moving particle with zero dimension. Rather it should be viewed as that of a photon whose EM field distribution is spread in local space.

We analyzed the orbital angular momentum (OAM) associated with an energy flow line. The OAM of a photon on a given trajectory is determined as a cross-product of a position vector and a linear momentum vector: $r\times \left(\hbar k\hat{P}\right)=-\hat{\theta }\hbar kr\frac{P_{\varphi }}{{\left(P_{r}^{2}+P_{\varphi }^{2}\right)}^{1/2}}$ , where $\hat{P}$ is a unit vector of Poynting vector $P=\hat{r}P_{r}+\hat{\varphi }P_{\varphi }$ (see Eq. (4) for ***P***). Figure 3D shows a sample trajectory plotted for *θ* = 90°. The OAM value, with reference to the origin at the dipole, is bounded between 0 and 2*ℏ*: 0 at *kr* = 0; *ℏ* at *kr* = 1.03; and 2*ℏ* at *kr* = ∞. Note that the asymptotic line drawn to the far-field trajectory is displaced a distance *d* of *λ*/π from the dipole: therefore, the OAM along this line remains constant at 2*ℏ*. Concerning the polar angle dependence at far field (*kr* >> 1), the OAM value takes a maximum (2*ℏ*) at *θ* = 90° and decreases to 0 at *θ* = 0 or 180°.

In our current formulation for OAM calculation, we postulate that the energy flow lines in far-fields are associated with a photon carrying momentum quanta (*ℏk*) along the energy flow direction ( $\hat{P}$ ). As a result of this quantum postulation, the OAM value in the above expression naturally takes the units of angular momentum quanta $\left(\right.\hbar$ ). By contrast, the SAM does not involve momentum $\left(\right.\hbar k$ ). Therefore, this formula cannot be applied to calculate SAM values [36].

From a quantum mechanical perspective, a hypothetical wave-function of a notional photon emanating from a dipole emitter can be viewed as a superposition of all plausible trajectories depicted by the energy flow distributions described above. The angular (*θ*, *φ*) dependence of Poynting vector distribution is $\hat{\varphi }\sin\theta$ at near field and $\hat{r}$ (cos^2^ *θ* + 1) at far field; and reveals a toroidal shape or a dumb-bell shape, respectively (Figure 3E and F). The arrows denote ***S*** and ***P*** vectors at select locations (Figure 3E); and at the locations where the likelihood of finding a photon is maximal (Figure 3F).

## 6 Chiral coupling and purcell enhancement

**Figure 4:**
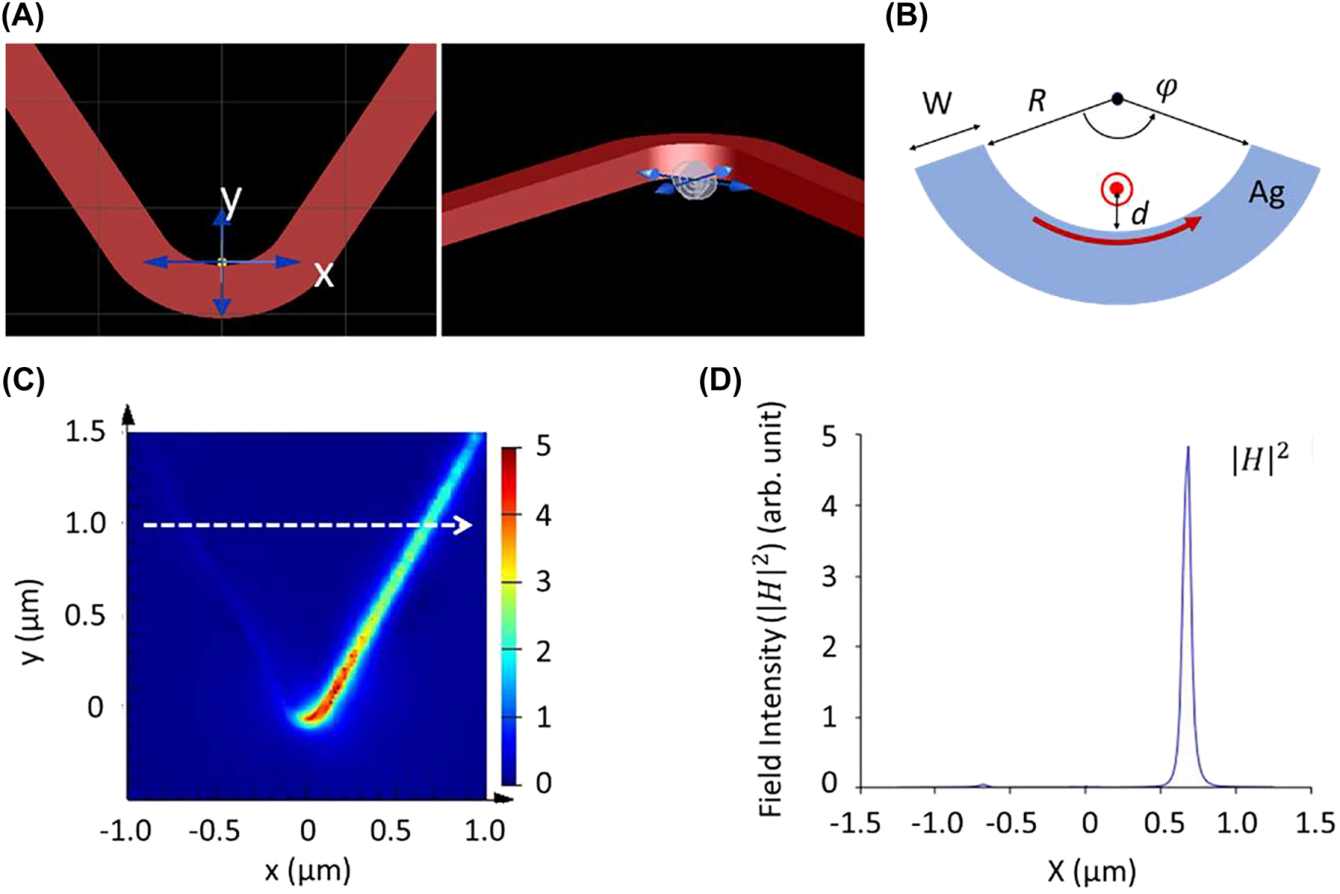
Chiral coupling of dipole field into surface plasmons. (A) Schematic of a plasmonic coupler. A left-circular-polarized dipole emitter is placed close to the bottom corner of the inner sidewall of a U-shape Ag waveguide (cross-section, 50 nm width and 50 nm height; arc angle of 120°). (B) The radius of curvature *R* (= 54 nm) of the circular part is designed to produce a curvature-induced OAM of $\hat{z}\hbar$ well matching the SAM of surface plasmons excited on metal surface; a dipole is placed at *d* = 3 nm from the sidewall. (C) Dipole field $\left(\left|H\right|\right)$ preferentially couples to the left arm of waveguide seen from the top (or to the right arm seen from the dipole). (D) SP field intensity $\left({\left|H\right|}^{2}\right)$ distributions measured after 1.16 µm propagation from the dipole. The polarization selectivity is estimated to be ∼113. This structure demonstrates Purcell enhancement of ∼17.

We investigated extracting the near-field energy flux to an outer world. We designed and simulated a plasmonic coupler structure for chiral extraction of dipole field energy into SPs: see Figure 4A and B for a U-shape Ag nanowire waveguide (cross-section, 50 nm width and 50 or 100 nm height). Here the radius of curvature *R* of a U-shape surface (the inner sidewall) is designed such that the OAM of an SP waveguide matches the SAM of an emitting photon [27, 28]: OAM = $r\times \left(\hbar k\hat{P}\right)=\pm \hat{z}\hbar k_{sp}R\equiv \pm \hat{z}\hbar$ = SAM, where $R=\frac{\lambda_{sp}}{2\pi }$ and *λ*_sp_ is the SP wavelength in the Ag waveguide. For *λ* = 633 nm, *λ*_sp_ is calculated to be 339 nm corresponding to *R* = 54 nm. In this design a circularly polarized dipole emitter (dipole axis along the *z*-direction) is located close to the waveguide surface: 3 nm distance from the inner sidewall and off from the corner in the diagonal direction. Being close to metal surface, a high-intensity energy flux can couple to SPs on a Ag waveguide (Supporting Information Section S5). This off-corner diagonal-configuration is to minimize image dipole formation: otherwise, this effect would cancel out the horizontal component of dipole moment and will result in a linearly polarized vertical dipole [37–42], adversely affecting chiral coupling. Poynting vectors of a linear dipole orient outward with a radially symmetric distribution and therefore are not suitable for chiral (asymmetric) coupling of dipole field.

From a phase matching perspective, i.e., the conservation of linear momentum along the tangential direction, the propagation direction of excited SPs should match the orientation of incident light’s Poynting vector (i.e., to the azimuthal direction: Figures 1D and 3E). In other words, dipole field must couple to the left-side waveguide (viewed from the dipole side) in this left-circular-polarized (LCP) dipole case. At the same time, angular momenta must be conserved as well. The *z*-component of the SAM vector projection to the inner sidewall of Ag waveguide has the following angular dependence: 1 − 3sin^2^ *θ*. Note that dipole spin vectors become orienting to the +*z*-direction for polar angle *θ* < 35° (Figures 1C and 3E): therefore, the angular momentum matching condition can be met only for those polar angles, and those photons will excite SPs propagating to the left-side waveguide. Speaking more specifically, the angular momentum conservation requirement must be met between incident photon spin and the curvature-induced OAM on local surface of Ag waveguide.

The coupling efficiency between an electric dipole and a waveguide mode is proportional to p* ⋅ ***E***, where ***p*** is the dipole moment and ***E*** is the electric field of the mode. The coupling efficiency takes a maximum value when ***p*** and ***E*** vectors are parallel to each other [43]. A similar relationship holds between the SAM vector SD of dipole field and the spin vector ***S***_sp_ of surface plasmons on metal surface, and the coupling efficiency is proportional to $S_{\text{d}}^{*}\cdot S_{\text{sp}}$ . SAM vector SD can be expressed referring to its rotating dipole moment ***p***_d_: $S_{\text{sp}}\propto Im\left(p_{\text{d}}^{*}\times p_{\text{d}}\right)$ . Similarly, surface plasmon spin vector ***S***_sp_ is $\propto Im\left(S_{\text{sp}}^{*}\times S_{\text{sp}}\right)$ . After some work, we obtain:

$$\begin{array}{ll} S_{d}^{*}\cdot S_{sp} & \propto \left\{\left[Re\left(p_{d}\right)\cdot Re\left(E_{sp}\right)\right]\left[Im\left(p_{d}\right)\cdot Im\left(E_{sp}\right)\right]\right.\\ & \;-\left.\left[Re\left(p_{d}\right)\cdot Im\left(E_{sp}\right)\right]\left[Im\left(p_{d}\right)\cdot Re\left(E_{sp}\right)\right]\right\} \end{array}$$

The dot product $S_{\text{d}}^{*}\cdot S_{\text{sp}}$ is found to take a maximum when the polarization vector ***p***_d_ of dipole field is parallel to the *E*-field of SP fields, basically the same relationship as the Fermi’s golden rule above.

A finite-difference time-domain (FDTD) simulation demonstrates chiral coupling (Figure 4C): The polarization selectivity is estimated to be ∼113 (Figure 4D). We also calculated Purcell enhancement of dipole emission [44–48]. Using a monitor box that surrounds a dipole emitter only, we measured the total power emanating from the dipole (through the box faces) for two different cases: with and without a Ag waveguide (Supporting Information Section S5). The ratio of the two powers corresponds to Purcell enhancement and is measured to be ∼17 at *d* = 3 nm. The importance of angular momentum matching for high-throughput chiral coupling is demonstrated by comparing different designs with widely varying curvature of metal surface (Figures S5 to S7).

## 7 Discussion

The large amount of energy flux trapped deep inside a near-field region corresponds to an electromagnetic energy flow associated with a rotating dipole moment and its electromagnetic field. The field-stored energy of dipole near-field is expressed as $\frac{e^{2}}{24\pi \varepsilon r_{1}}$ , where *r*_1_ is the lower limit of volume integral of energy density and dipole moment *p*_0_ is assumed to be *r*_1_e (Supporting Information Section S6). Assuming *r*_1_ = 1 nm, the electromagnetic energy is calculated to be 4 × 10^−20^ J: this amount corresponds to 12% of the photon energy at the assumed wavelength (*ℏω* = 1.96 eV for *λ* = 633 nm). Moreover, this energy expression is found to hold the following relationship with the trapped energy flux derived above: the near-field energy flux equals a product of oscillation frequency (*ω*) and field-stored energy (Supporting Information Section S6). This supports the notion that the large energy flux is a manifestation of field-stored energy that is spinning fast at optical frequency.

The photon distribution analyzed in this work resembles the electron cloud model of an atom, in which the angular (*θ*, *φ*) dependence of probability density corresponds to eigenfunctions of Schrödinger equation for hydrogen atom and are expressed as spherical harmonics specified with two quantum numbers (orbital *l* and magnetic *m*_*l*_). Similar to electron orbitals, photons reveal an orbital structure whose angular momentum (AM) values are bounded to an integer multiple of *ℏ*: 2*ℏ* for OAM; and *ℏ* for total AM. Here the quantum nature of both cases (discreteness of angular momenta) is ascribed to the compactness of sphere on which photons and electron wavefunctions are defined. The near-field-trapped photonic orbitals ( $\hat{\varphi }\sin\theta$ ) revealed in the present work (Figure 3E) can be viewed as a possible electromagnetic model that may explain the non-radiating nature of the electron orbitals associated with the ground state of a stable atom [49–51].

With a plasmonic coupler, SP losses might be significant [52]. SP propagation loss, however, can be alleviated to an insignificant level by reducing the length of a metal waveguide section to minimum and by transfer coupling of SPs into a low-loss dielectric waveguide [47]. A high-efficiency chiral coupler structure with high polarization-selectivity and Purcell factor is expected to play an important role in developing single photon source chips that offer high rate of generation and transmission of photon qubits [53–55].

## Supporting Information

Detailed derivations, notes and numerical analysis/simulation results (PDF).

## Supplementary Material

Supplementary Material Details
