# Supplementary material for: Spin texture and chiral coupling of circularly polarized dipole field
Source: Nanophotonics. 2023 Jan 3;12(1):129–38. doi: 10.1515/nanoph-2022-0581 (PMC11501285; doi:10.1515/nanoph-2022-0581)
Supplement: Supplementary file 1 — Supplementary Material Details [file j_nanoph-2022-0581_suppl.pdf]

Supporting Information

Spin texture and chiral coupling of circularly-polarized dipole field

Yu Shi and Hong Koo Kim

Correspondence to: hkk@pitt.edu

Supporting Information Section, S1 to S6
Figs. S1 to S7
References S51 & S52

Supporting Information Section

S1. Derivation of spin angular momentum (SAM) density of a circularly polarized electric dipole: the contributions by electric- and magnetic-fields

The electric and magnetic fields of an electric point dipole are expressed in SI units as follows [29].

$$\boldsymbol{E}(\boldsymbol{r}) = \frac{1}{4\pi\varepsilon} e^{ik\cdot r}\left[(\hat{\boldsymbol{r}} \times \boldsymbol{p}) \times \hat{\boldsymbol{r}}\,\frac{k^2}{r} + (3\hat{\boldsymbol{r}}(\hat{\boldsymbol{r}} \cdot \boldsymbol{p}) - \boldsymbol{p})\left(\frac{1}{r^3} - \frac{ik}{r^2}\right)\right]$$

$$\boldsymbol{H}(\boldsymbol{r}) = \frac{1}{4\pi\sqrt{\mu\varepsilon}} e^{ik\cdot r}\left[(\hat{\boldsymbol{r}} \times \boldsymbol{p})\,\frac{k^2}{r}\left(1 - \frac{1}{ikr}\right)\right]$$

Consider the case of dipole moment $\boldsymbol{p} = \frac{p_0}{\sqrt{2}}(\hat{\boldsymbol{x}} + i\hat{\boldsymbol{y}})$ that is located at the origin of coordinates, and whose polarization vector lies on the *x*-*y* plane and rotates around the dipole axis (the *z*-axis).

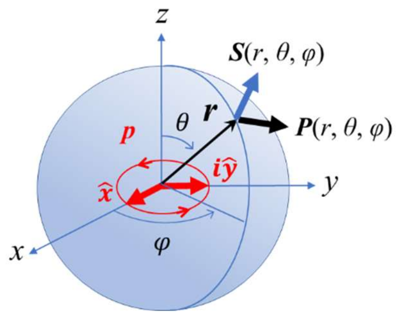

Fig. S1. A circularly polarized electric dipole $\boldsymbol{p} = \frac{p_0}{\sqrt{2}}(\hat{\boldsymbol{x}} + i\hat{\boldsymbol{y}})$ is placed at the origin of coordinates. $\boldsymbol{S}$ and $\boldsymbol{P}$ denote the resulting spin angular momentum (SAM) density and Poynting vector, respectively at an observation point $\boldsymbol{r}$.

Let's express the *E*- and *H*-field in spherical coordinates. We apply the following relationship between the unit vectors of Cartesian coordinates (*x, y, z*) and those of spherical coordinates (*r*, θ, φ).

$$\hat{\boldsymbol{x}} = \sin\theta\,\cos\varphi\,\hat{\boldsymbol{r}} + \cos\theta\,\cos\varphi\,\hat{\boldsymbol{\theta}} - \sin\varphi\,\hat{\boldsymbol{\varphi}}$$

$$\hat{\boldsymbol{y}} = \sin\theta\,\sin\varphi\,\hat{\boldsymbol{r}} + \cos\theta\,\sin\varphi\,\hat{\boldsymbol{\theta}} + \cos\varphi\,\hat{\boldsymbol{\varphi}}$$

$$\hat{\boldsymbol{r}} \times \boldsymbol{p} = p_0 e^{i\varphi}\left(\cos\theta\, \hat{\boldsymbol{\varphi}} - i\, \hat{\boldsymbol{\theta}}\right)$$

$$\hat{\boldsymbol{r}} \cdot \boldsymbol{p} = p_0 e^{i\varphi} \sin\theta$$

We obtain the following expressions of electric and magnetic fields.

$$\boldsymbol{E}(\boldsymbol{r}) = \frac{1}{4\pi\varepsilon}\frac{p_0}{\sqrt{2}} e^{i\boldsymbol{k}\cdot\boldsymbol{r}} e^{i\varphi}\left[2\left(\frac{1}{r^3} - \frac{ik}{r^2}\right)\sin\theta\, \hat{\boldsymbol{r}} + \left(\frac{k^2}{r} + \frac{-1}{r^3} + \frac{ik}{r^2}\right)\left(\cos\theta\, \hat{\boldsymbol{\theta}} + i\, \hat{\boldsymbol{\varphi}}\right)\right]$$

$$\boldsymbol{H}(\boldsymbol{r}) = \frac{1}{4\pi\sqrt{\mu\varepsilon}}\frac{p_0}{\sqrt{2}} e^{i\boldsymbol{k}\cdot\boldsymbol{r}} e^{i\varphi}\left(\frac{k^2}{r} + \frac{ik}{r^2}\right)\left(-i\, \hat{\boldsymbol{\theta}} + \cos\theta\, \hat{\boldsymbol{\varphi}}\right)$$

Spin angular momentum (SAM) density $\boldsymbol{S}$ is defined as

$$\boldsymbol{S}(\boldsymbol{r}) = \frac{1}{4\omega}\left[\varepsilon \cdot \mathrm{Im}(\boldsymbol{E}^* \times \boldsymbol{E}) + \mu \cdot \mathrm{Im}(\boldsymbol{H}^* \times \boldsymbol{H})\right]$$

The E-field contribution to $\boldsymbol{S}$ is determined as follows.

$$\boldsymbol{E}^* \times \boldsymbol{E} = i\frac{1}{(4\pi\varepsilon)^2} p_0^2\left[\left(\frac{1}{r^6} - \frac{k^2}{r^4} + \frac{k^4}{r^2}\right)\cos\theta\, \hat{\boldsymbol{r}} + \frac{2}{r^6}\sin\theta\, \hat{\boldsymbol{\theta}} + \frac{k^3}{r^3}\sin 2\theta\, \hat{\boldsymbol{\varphi}}\right]$$

$$\boldsymbol{S_E} = \frac{1}{4\omega}\varepsilon\, \mathrm{Im}(\boldsymbol{E}^* \times \boldsymbol{E}) = \frac{1}{4\omega}\frac{1}{(4\pi)^2}\frac{1}{\varepsilon} p_0^2\left[\left(\frac{1}{r^6} - \frac{k^2}{r^4} + \frac{k^4}{r^2}\right)\cos\theta\, \hat{\boldsymbol{r}} + \frac{2}{r^6}\sin\theta\, \hat{\boldsymbol{\theta}} + \frac{k^3}{r^3}\sin 2\theta\, \hat{\boldsymbol{\varphi}}\right]$$

Similarly, the H-field contribution to $\boldsymbol{S}$ is determined as follows.

$$\boldsymbol{H}^* \times \boldsymbol{H} = i\frac{1}{(4\pi)^2}\frac{1}{\mu\varepsilon} p_0^2\left(\frac{k^4}{r^2} + \frac{k^2}{r^4}\right)\cos\theta\, \hat{\boldsymbol{r}}$$

$$\boldsymbol{S_H} = \frac{1}{4\omega}\mu\, \mathrm{Im}(\boldsymbol{H}^* \times \boldsymbol{H}) = \frac{1}{4\omega}\frac{1}{(4\pi)^2}\frac{1}{\varepsilon} p_0^2\left(\frac{k^4}{r^2} + \frac{k^2}{r^4}\right)\cos\theta\, \hat{\boldsymbol{r}}$$

The total SAM density $\boldsymbol{S}$ is determined as

$$\boldsymbol{S} = \boldsymbol{S_E} + \boldsymbol{S_H} = \frac{1}{4\omega}\frac{1}{(4\pi)^2}\frac{1}{\varepsilon} p_0^2\left[\left(\frac{1}{r^6} + \frac{2k^4}{r^2}\right)\cos\theta\, \hat{\boldsymbol{r}} + \frac{2}{r^6}\sin\theta\, \hat{\boldsymbol{\theta}} + \frac{k^3}{r^3}\sin 2\theta\, \hat{\boldsymbol{\varphi}}\right]$$

Note that the H-field is comprised of $1/r^2$ and $1/r^4$ terms but not $1/r^6$ term.

This results in zero contribution to spin density $\boldsymbol{S}$ at near field; cancellation of the E-field contribution (the $1/r^4$ term) at intermediate field; and equal contribution (the $1/r^2$ term) as the E-field at far field.

S2. Calculation of the total energy flux of a near-field concentrated wave of a circularly polarized dipole

An energy flow density (time-averaged Poynting vector) $\boldsymbol{P}$ is defined as ½ Re($\boldsymbol{E}^*$ x $\boldsymbol{H}$). Referring to the expressions of E- and H-fields derived above, an energy flow density $\boldsymbol{P}$ is determined as follows.

$$\boldsymbol{P}(\boldsymbol{r}) = \frac{1}{(4\pi)^2}\frac{1}{\varepsilon\sqrt{\mu\varepsilon}}\frac{p_0^2}{4}\left[\frac{k^4}{2r^2}(3+\cos 2\theta)\,\hat{\boldsymbol{r}} + 2\left(\frac{k^3}{r^3}+\frac{k}{r^5}\right)\sin\theta\,\hat{\boldsymbol{\varphi}}\right]$$

At near field, we can keep the $1/r^5$ term only: the resulting energy flow direction becomes azimuthal, that is, circling around the dipole axis. This energy flux density is determined as

$$\boldsymbol{P}(\boldsymbol{r}) = \frac{1}{2}\frac{1}{(4\pi)^2}\frac{1}{\varepsilon\sqrt{\mu\varepsilon}}p_0^2\frac{k}{r^5}\sin\theta\,\hat{\boldsymbol{\varphi}}$$

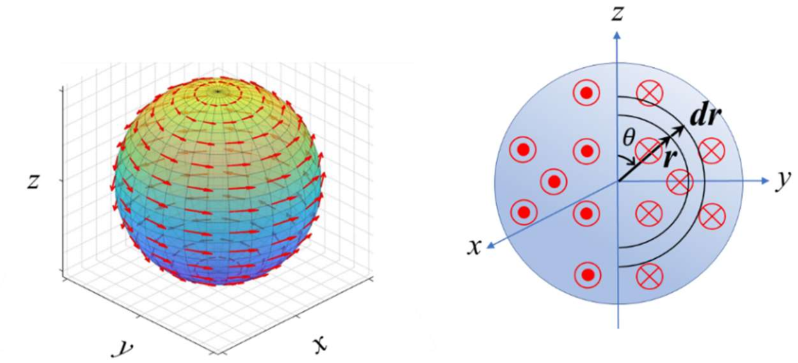

Fig. S2. Calculation of a near-field saved energy flux orbiting around the dipole axis of a circularly polarized electric dipole. (left) Poynting vectors at near field ($kr \ll 1$) orient to the azimuthal direction. (right) The total flux of this confined energy flow is calculated by integrating over for
r (r_1 to r_2; $r_1 \ll r_2$) and θ (0 to π).

The total energy flux is calculated by integrating over for r and θ: r from r_1 to r_2; θ from 0 to π.

$$\int \boldsymbol{P}(\boldsymbol{r})\cdot \mathrm{d}\boldsymbol{A} = \int_{\boldsymbol{\theta=0}}^{\boldsymbol{\pi}}\int_{\boldsymbol{r=r1}}^{\boldsymbol{r2}} \boldsymbol{P}(\boldsymbol{r})\cdot\hat{\boldsymbol{\varphi}}\,r dr d\theta = \frac{1}{(4\pi)^2}\frac{1}{\varepsilon\sqrt{\mu\varepsilon}}p_0^2\frac{k}{3}\left(\frac{1}{r_1^3}-\frac{1}{r_2^3}\right)$$

Here we assume $r_1 << r_2$. Then the total energy flux becomes.

$$\int \boldsymbol{P}(\boldsymbol{r}) \cdot \mathrm{d}\boldsymbol{A} = \frac{1}{3}\frac{1}{(4\pi)^2}\frac{1}{\varepsilon\sqrt{\mu\varepsilon}}p_0^2\frac{k}{r_1^3} = \frac{ck}{48\pi^2\varepsilon}\frac{p_0^2}{r_1^3}$$

S3. Calculation of the total radiation power of a circularly polarized dipole

Refer to the Poynting vector expression given above (S2). For far-field analysis, we can keep the $1/r^2$ term only. An energy flow density at far field is given as

$$\boldsymbol{P}(\boldsymbol{r}) = \frac{1}{4}\frac{1}{(4\pi)^2}\frac{1}{\varepsilon\sqrt{\mu\varepsilon}}p_0^2\frac{k^4}{r^2}(1+\cos^2\theta)\,\hat{\boldsymbol{r}}$$

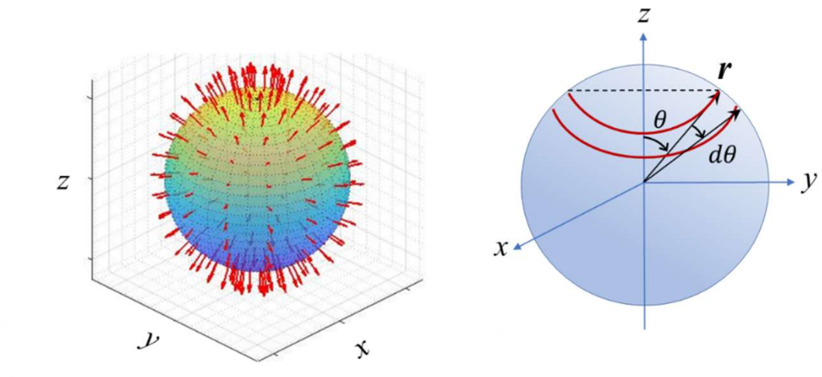

Fig. S3. Calculation of total radiation power of circularly polarized dipole field. (left) Poynting vectors at far field orient to the radial direction. (right) Total radiation power is calculated by integrating over the entire surface of sphere (θ, φ).

The total radiation flux is obtained by integrating for θ from 0 to π.

$$\oint \boldsymbol{P}(\boldsymbol{r}) \cdot \mathrm{d}\boldsymbol{A} = \int_{\boldsymbol{\theta}=\boldsymbol{0}}^{\boldsymbol{\pi}} \boldsymbol{P}(\boldsymbol{r}) \cdot \hat{\boldsymbol{r}}\; 2\pi r^2 \sin\theta\; d\theta = \frac{ck^4}{12\pi\varepsilon}p_0^2.$$

Assume dipole moment p_0 = [1 (nm)] x [1.6x10^{-19} (C)]; λ = 633 nm; and free space ($\varepsilon = \varepsilon_0$). The total radiation flux is calculated to be 2.2x10^{-10} J or 7x10^{8} photons per second. This corresponds to emission lifetime of 1.4 ns.

S4. Calculation of the winding number of an energy flow line on an equatorial plane (θ = 90 deg).

Consider the case that an energy flow line emanates from a circularly polarized electric dipole and radiates away on the equatorial plane (θ = 90 deg).

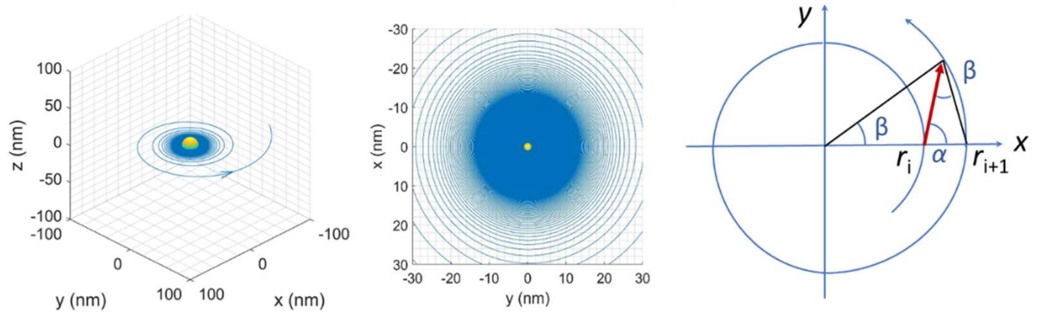

Fig. S4. Calculation of the number of windings of an energy flow line on an equatorial plane (θ = 90 deg)

The Poynting vector ***P*** at the *x*-axis intercept corresponds to a tangential vector to the energy flow line at that point. ***P*** vector lies on the (r, φ)-plane and can be expressed as $\boldsymbol{P} = P_r\hat{\boldsymbol{r}} + P_\varphi\hat{\boldsymbol{\varphi}}$. At near field, $P_r << P_\varphi$ and the following relationship holds: see Fig. S6.

$$\alpha + \frac{1}{2}\beta = \frac{\pi}{2}\ ;\ \tan\alpha = \frac{P_\varphi}{P_r}$$
$$\frac{P_r}{P_\varphi} = \tan\left(\frac{1}{2}\beta\right) \cong \frac{1}{2}\beta$$

The arc length for incremental angle β and radius r is $r\beta$.
The interval (dr) between two neighboring intercepts on the horizontal axis is

$$\mathrm{d}r = r_{i+1} - r_i = r\beta^2 = r\left(2\frac{P_r}{P_\varphi}\right)^2 = 4r\left(\frac{P_r}{P_\varphi}\right)^2$$

Referring to the Poynting vector expression derived above (S2), the P_r/P_φ ratio at θ = 90 deg is determined as follows. We also assume a near-field case, i.e., $kr << 1$.

$$\frac{P_r}{P_\varphi} = \frac{\frac{k^4}{r^2}}{\frac{2k^3}{r^3} + \frac{2k}{r^5}} = \frac{(kr)^3}{2(kr)^2 + 2} \cong \frac{(kr)^3}{2}$$

The interval dr is determined as: $\mathrm{d}r = r(kr)^6$.
The number of windings (NW) per unit distance is: $\mathrm{NW} = \frac{1}{\mathrm{d}r} = \frac{1}{r(kr)^6} = \frac{k}{(kr)^7}$

The total number of windings (TNW) in the radial distance r in the range from r_1 to r_2 is determined as:

$$TNW = \int_{r1}^{r2} \frac{k}{(kr)^7} dr = \frac{1}{6}\left[\frac{1}{(kr_1)^6} - \frac{1}{(kr_2)^6}\right]$$

Assume $r_2 >> r_1$:

$$TNW = \int_{r1}^{r2} \frac{k}{(kr)^7} dr \cong \frac{1}{6(kr_1)^6}$$

Below is a summary of sample calculations of the total number of windings for the radial distance in the near-field regime (r_1 = 1 - 10 nm) and at λ = 633 nm in free space.

r_1 (nm)	Total number of windings (TNW)
1	1.74×10^{11}
3	2.39×10^{8}
5	1.12×10^{7}
10	1.74×10^{5}

S5. Numerical analysis of surface plasmon excitation in Ag nanowire waveguides

Finite-difference time-domain (FDTD) simulation was carried out by using Lumerical software. The following refractive index values are assumed for Ag and SiO2 at 633-nm wavelength.

λ_0	Ag	SiO2 (fused silica)
0.633μm	n = 0.0562 + 4.2776i [S56]	n = 1.4570 [S57]

The following three configurations and waveguide geometries were simulated and compared.

Configuration 1:
A U-shape Ag waveguide (radius of curvature R = 54 nm; 50-nm width and 50-nm height; arc angle of 60 deg) with a circularly-polarized dipole placed at distance d = 3 nm from the inner sidewall and close to the bottom corner

A dipole emitter is placed next to waveguide surface: 3-nm distance from the inner sidewall and off from the bottom corner in the diagonal direction. The dipole axis orients to the z-direction. A monitor box (yellow; cube with 3-nm side length) is placed enclosing the dipole only and excluding the Ag waveguide. The total energy flux emanating from the dipole is calculated with and without the Ag waveguide. Purcell factor is calculated by taking the ratio of the two powers.

For a left-circular polarized dipole, excited SPs propagate to the right-side branch seen from the top (to the left-side branch seen from the dipole).

- Polarization selectivity is measured to be 113: the ratio of the magnetic field intensity $|H|^2$ measured after 1.16 μm propagation from the dipole emitter.
- Purcell factor is measured to be 17: the ratio of the two powers emanating from a monitor box.

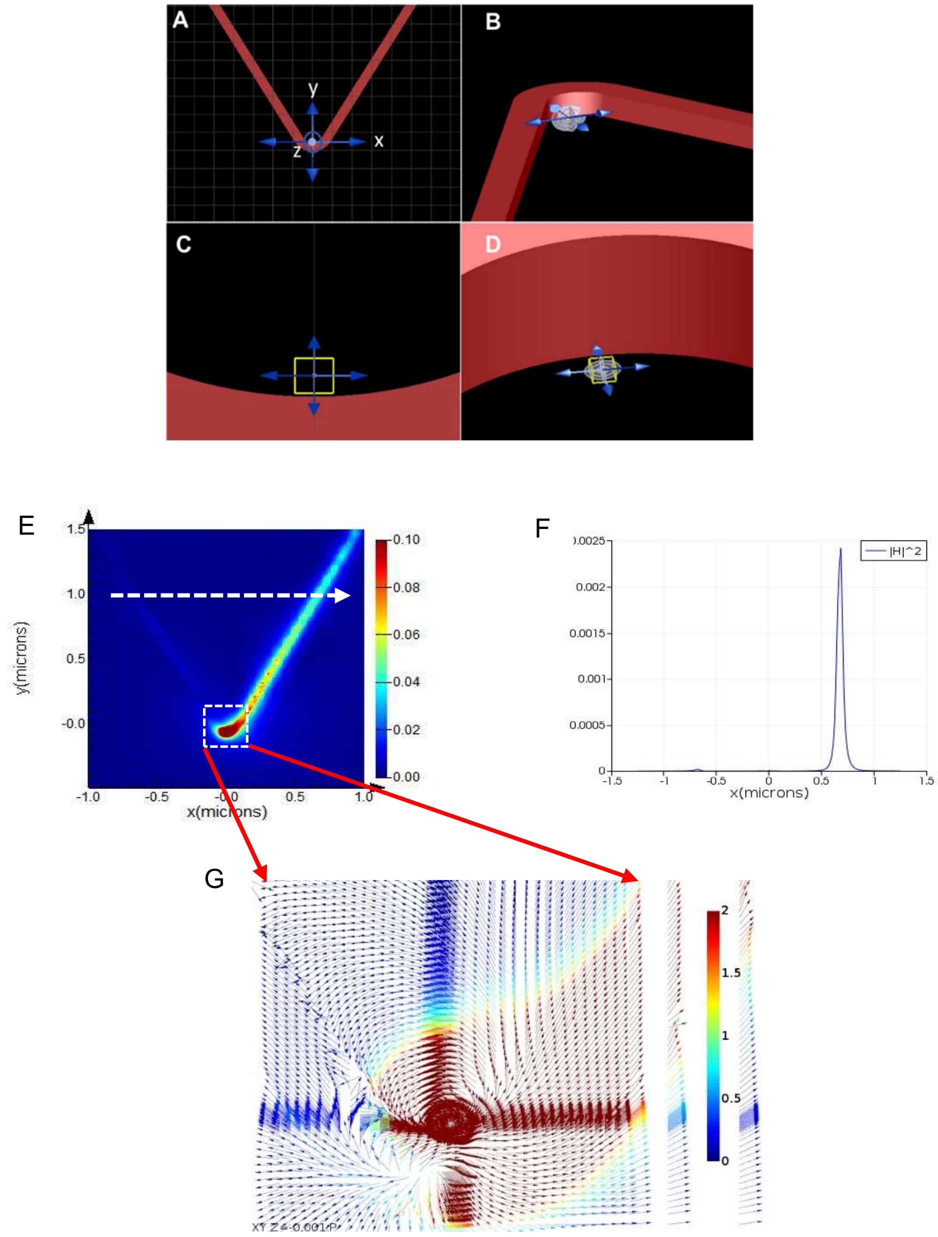

Fig. S5. (A to D) Schematic of a U-shape Ag waveguide for chiral coupling of dipole field. A dipole is placed close to the bottom corner of a Ag waveguide (d = 3 nm). (E) The H-field

distribution: top view at 1-nm above Ag waveguide top surface. (F) The H-field intensity profile scanned after 1.16 µm propagation from a dipole. (G) Poynting vector distribution around the dipole emitter.

<u>**Configuration 2**:</u>

<u>A U-shape Ag waveguide (radius of curvature R = 54 nm; 50-nm width and 50-nm height; arc angle of 60 deg) with a circularly-polarized dipole placed at the center of circle ($d = R$ = 54 nm) and close to the bottom corner</u>

- Polarization selectivity is measured to be 7: the ratio of the magnetic field intensity $|H|^2$ measured after 1.16 µm propagation from the dipole emitter.
- Purcell factor is measured to be 2.4: the ratio of the two powers emanating from a monitor box.

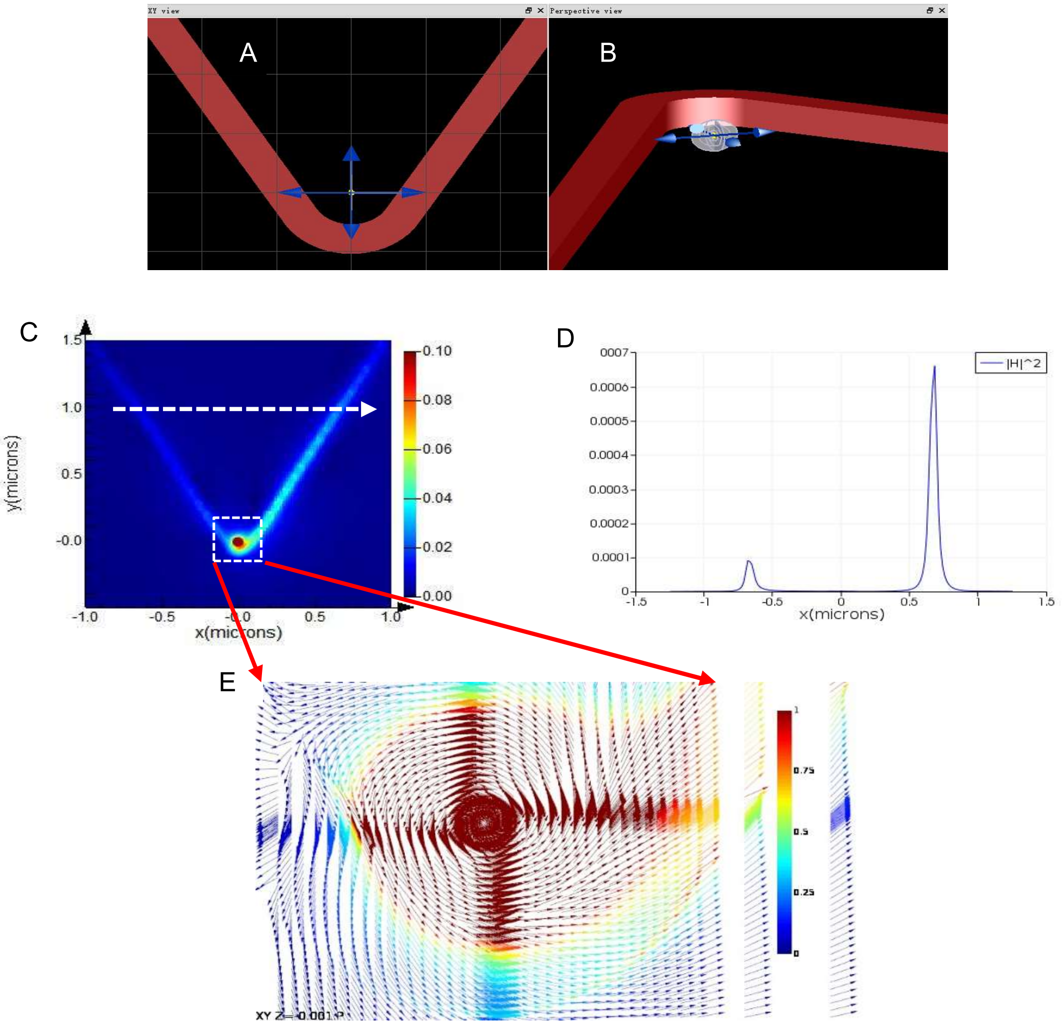

Fig. S6. (A & B) Schematic drawing of a U-shape Ag waveguide for chiral coupling of dipole field. A dipole is placed at the center of circle: $d = R$ = 54 nm. (C) The H-field distribution: top

view at 1-nm above Ag waveguide top surface. (D) The H-field intensity profile scanned after 1.16 µm propagation. (E) Poynting vector distribution around the dipole emitter.

<u>Configuration 3</u>:
<u>A linear straight Ag waveguide (50-nm width and 50-nm height) with a circularly-polarized dipole placed at distance $d = 3$ nm from the inner sidewall and close to the bottom corner</u>

- Polarization selectivity is measured to be 5.5: the ratio of the magnetic field intensity $|H|^2$ measured after 1.16 µm propagation from the dipole emitter.
- Purcell factor is measured to be 18: the ratio of the two powers emanating from a monitor box.

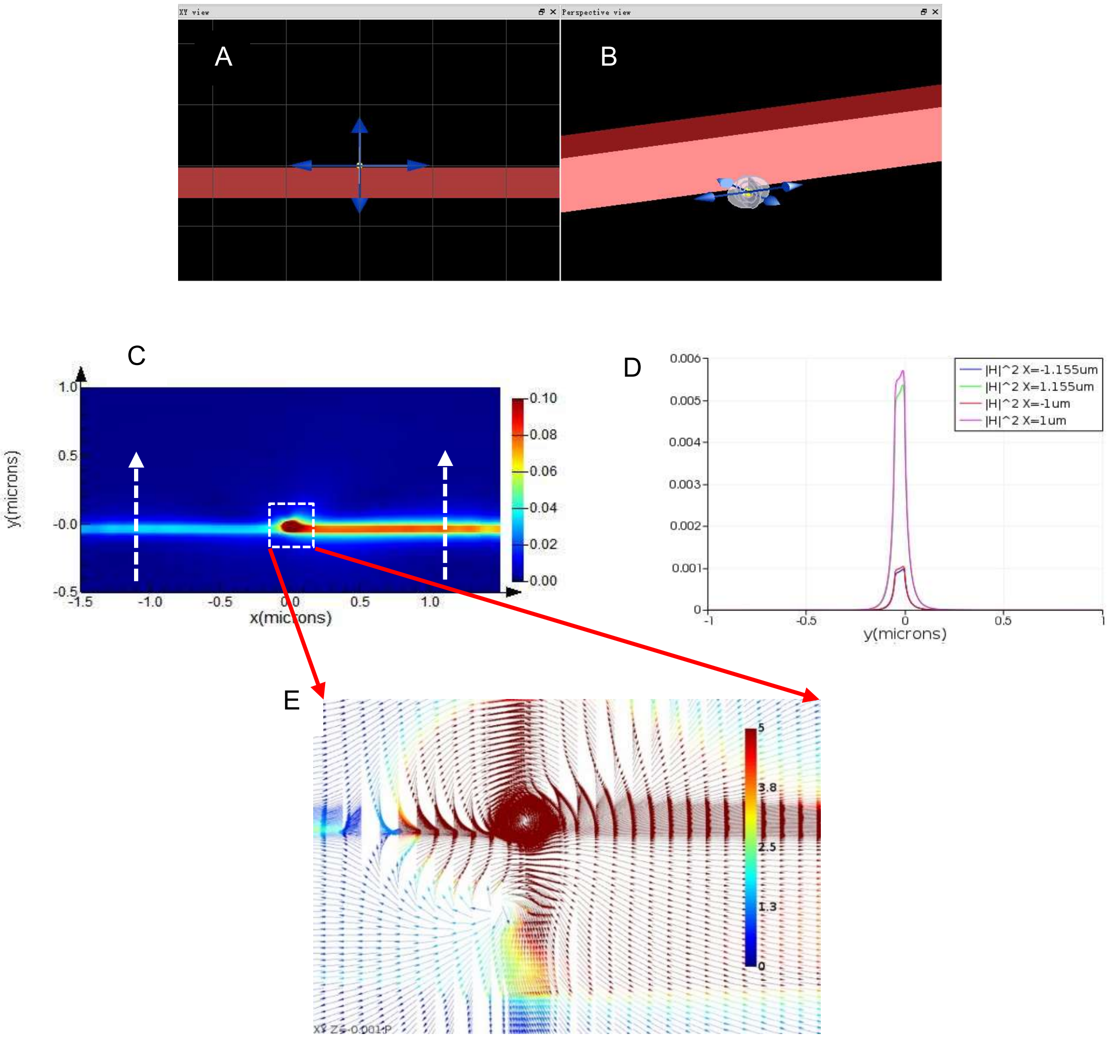

Fig. S7. (A & B) Schematic drawing of a linear Ag waveguide for chiral coupling of dipole field. (C) The H-field distribution: top view at 1-nm above Ag waveguide top surface. (D) The H-field

intensity profile scanned after 1.16 μm propagation. (E) Poynting vector distribution around the dipole emitter.

S6. Derivation of field-stored energy of a circularly polarized electric dipole

The electromagnetic energy density is given by $u = \frac{1}{2}(\boldsymbol{E} \cdot \boldsymbol{D} + \boldsymbol{B} \cdot \boldsymbol{H})$.
Let's assume harmonic fields and take the near-field term only from the ***E***- and ***H***-field expressions derived in S1.

The ***E***-field stored energy density u_E is determined as

$$u_\mathrm{E} = \frac{1}{4}\boldsymbol{E} \cdot \boldsymbol{D}^* = \frac{1}{128\pi^2\varepsilon}\frac{p_0^2}{r^6}(3\sin^2\theta + 2)$$

The ***H***-field stored energy density u_H is determined as

$$u_\mathrm{H} = \frac{1}{4}\boldsymbol{B} \cdot \boldsymbol{H}^* = \frac{\omega^2\mu}{128\pi^2}\frac{p_0^2}{r^4}(\cos^2\theta + 1)$$

The total *E*-field energy is determined as

$$w_\mathrm{E} = \iint u_E dV = \iint_{r=r_1;\,\theta=0}^{r=\infty;\,\theta=\pi} u_\mathrm{E}\, 2\pi r^2 \sin\theta dr d\theta = \frac{1}{24\pi\varepsilon}\frac{p_0^2}{r_1^3}$$

The total *H*-field energy is determined as

$$w_\mathrm{H} = \iint u_\mathrm{H} dV = \iint_{r=r_1;\,\theta=0}^{r=\infty;\,\theta=\pi} u_\mathrm{H}\, 2\pi r^2 \sin\theta \mathrm{d}r\mathrm{d}\theta = \frac{\omega^2\mu}{24\pi}\frac{p_0^2}{r_1}$$

The ratio of the two field-stored energies is

$$\frac{w_\mathrm{E}}{w_H} = \frac{1}{(kr_1)^2}$$

At near field ($kr_1 << 1$), the *H*-field energy is negligible and the total energy becomes

$$w = w_\mathrm{E} = \frac{1}{24\pi\varepsilon}\frac{p_0^2}{r_1^3}$$

Let's assume dipole moment $p_0 = r_1 e$. The total energy can be expressed as

$$w = \frac{e^2}{24\pi\varepsilon r_1}$$

Let’s compare this field-stored energy with the energy flux derived before (see S2).

$$\int \boldsymbol{P}(\boldsymbol{r}) \cdot \mathrm{d}\boldsymbol{A} = \frac{\omega}{48\pi^2\varepsilon} \frac{p_0^2}{r_1^3} = f \cdot w_\mathrm{E}$$

[trapped energy flux] = [frequency] x [field-stored energy].

References

29. J. D. Jackson, *Classical Electrodynamics* (Wiley, New York, ed. 3, 1999).

S56. P. B. Johnson and R. W. Christy, “Optical constants of the noble metals,” *Phys. Rev. B* 6, 4370-4379 (1972).

S57. I. H. Malitson, “Interspecimen comparison of the refractive index of fused silica,” *J. Opt. Soc. Am.* 55, 1205-1208 (1965).
